# Salicylic Acid in Rheumatism

**Published:** 1876-09

**Authors:** 


Salicylic Acid in Rheumatism.—At the time of the appearance in the *Medical Record* of the first article relating to the use of salicylic acid in acute inflammatory rheumatisn, I had in charge a patient suffering with that "most unkindest" affection.

Mr. M., aged 28 years, a healthy working man, of good habits, and employed in the public school buildings, was seized suddenly and rather violently on the morning of April 18. The attack was ushered in with the usual symptoms; and at the time I concluded to try the salicylic acid the disease was pursuing the even tenor of its way, having reached the eleventh day. At this time the articulations in the limbs and upper extremities were greatly swollen, intensely red, and very painful. The patient was helpless, and it was with great difficulty that he could be moved.

A vigorous alkaline treatment, with quinine and colchicum during the latter part of the time, had resulted in no apparent benefit. Perspiration was profuse, the urine alkaline in its reaction, and the patient very much prostrated. The pain was intense and constant, except when under the influence of an anodyne. I ordered the acid given in five-grain doses every hour, the vehicle being equal parts of glycerine and water. After the sixth dose I noticed a marked reduction in temperature. The extremities became cold, the circulation thready, and there seemed to be an entire loss of memory. These conditions rapidly gave way to stimulation, however, and in fifteen hours after the administration of the first dose the pain and swelling had entirely disappeared. On the second day after beginning the acid treatment the patient took his dinner at the table, being able to feed himself. Treatment was continued for two days longer, the doses being given at intervals of three hours. The patient made a good recovery, there being no return of the disease.

On May 17 another case of equal interest to me came under my observation. Mr. E., aged 22, a plasterer by trade, was taken with acute inflammatory rheumatism, affecting the upper extremities. Two years ago this patient had a similar attack, and the disease gradually extended until most of the articulations of the body were affected. The usual treatment was given, but the case continued twenty-six days, and I had but negative evidence that my treatment had been of any avail. When I called to see him during this last attack I was met with: "Doctor, I'm gone up again for twenty-six days." He was suffering intensely, and I at once gave him $\frac{1}{4}$ gr. morphia subcutaneously, after which I ordered the acid as before. In this case the reduction of temperature was not so marked, and there was an absence of the unpleasant symptoms noted in the first case. The result, however, was all that could be desired. In twenty hours the pain and swelling had disappeared, and in one week the patient was pursuing his usual avocation. In this case no medicine whatever was given except acid—save the $\frac{1}{4}$ gr. morphia used subcutaneously.—*Medical Record.*

